# Optimized recombinant production of the bacteriocin garvicin Q by *Corynebacterium glutamicum*

**DOI:** 10.3389/fmicb.2023.1254882

**Published:** 2024-01-08

**Authors:** Christian K. Desiderato, Carolin Müller, Alexander Schretzmeier, Katharina M. Hasenauer, Bruno Gnannt, Bastian Süpple, Alexander Reiter, Valentin Steier, Marco Oldiges, Bernhard J. Eikmanns, Christian U. Riedel

**Affiliations:** ^1^Institute of Molecular Biology and Biotechnology of Prokaryotes, University of Ulm, Ulm, Germany; ^2^Institute of Bio- and Geosciences, IBG-1: Biotechnology, Forschungszentrum Jülich GmbH, Jülich, Germany; ^3^Institute of Biotechnology, RWTH Aachen University, Aachen, Germany

**Keywords:** *Corynebacterium glutamicum*, bacteriocin, recombinant production, Sec-dependent secretion, HtrA protease

## Abstract

Bacteriocins are antimicrobial peptides applied in food preservation and are interesting candidates as alternatives to conventional antibiotics or as microbiome modulators. Recently, we established *Corynebacterium glutamicum* as a suitable production host for various bacteriocins including garvicin Q (GarQ). Here, we establish secretion of GarQ by *C. glutamicum* via the Sec translocon achieving GarQ titers of about 7 mg L^–1^ in initial fermentations. At neutral pH, the cationic peptide is efficiently adsorbed to the negatively charged envelope of producer bacteria limiting availability of the bacteriocin in culture supernatants. A combination of CaCl_2_ and Tween 80 efficiently reduces GarQ adsorption to *C. glutamicum*. Moreover, cultivation in minimal medium supplemented with CaCl_2_ and Tween 80 improves GarQ production by *C. glutamicum* to about 15 mg L^–1^ but Tween 80 resulted in reduced GarQ activity at later timepoints. Using a reporter strain and proteomic analyses, we identified HtrA, a protease associated with secretion stress, as another potential factor limiting GarQ production. Transferring production to HtrA-deficient *C. glutamicum* K9 improves GarQ titers to close to 40 mg L^–1^. Applying conditions of low aeration prevented loss in activity at later timepoints and improved GarQ titers to about 100 mg L^–1^. This is about 50-fold higher than previously shown with a *C. glutamicum* strain employing the native GarQ transporter GarCD for secretion and in the range of levels observed with the native producer *Lactococcus petauri* B1726. Additionally, we tested several synthetic variants of GarQ and were able to show that exchange of the methionine in position 5 to a phenylalanine (GarQ*^M5F^*) results in markedly increased activity against *Lactococcus lactis* and *Listeria monocytogenes*. In summary, our findings shed light on several aspects of recombinant GarQ production that may also be of relevance for production with natural producers and other bacteriocins.

## 1 Introduction

Bacteriocins are ribosomally synthesized antimicrobial peptides or proteins that kill sensitive bacteria or inhibit their growth (Darbandi et al., 2022). Bacteriocins of lactic acid bacteria (LAB), especially model peptides like nisin and pediocin, have been widely studied (Chikindas et al., 2018; Silva et al., 2018) and are classified into modified (class I), non-modified (class II) peptides and thermo-labile proteins (class III) (Alvarez-Sieiro et al., 2016; Acedo et al., 2018). A more recent review adds a class IV with lipid or carbohydrate moieties to the classification (Darbandi et al., 2022). While the majority of the known LAB bacteriocins are secreted by dedicated ABC-transporters (Nes et al., 1996; Zheng and Sonomoto, 2018), there are examples of class II bacteriocins (e.g., divergicin A or enterocin P) that are secreted by the general secretory (Sec) pathway by their native producers (Worobo et al., 1995; Nes et al., 1996; Herranz and Driessen, 2005). Depending on the secretion pathway, different N-terminal signal peptides (SPs) are required guiding the prepeptide to the transporter. After or during translocation, the SP is cleaved by a signal peptidase and the active peptide is released (Nes et al., 1996; Zheng and Sonomoto, 2018). In case of dedicated ABC-transporters, cleavage occurs directly after a double-glycine-motif in the SP (Nes et al., 1996; Zheng and Sonomoto, 2018). In contrast, Sec-dependent SPs have a tripartite structure with a positively charged N-terminal part, a hydrophobic central region and a polar C-terminal region containing the conserved AXA recognition site for signal peptidase cleavage (Freudl, 2018). Interestingly, double-glycine-type and Sec-dependent SPs can be exchanged, and it was demonstrated that the Sec pathway can be employed for heterologous bacteriocin production (McCormick et al., 1996; Van Belkum et al., 1997; Martín et al., 2007; Borrero et al., 2011; Feito et al., 2023).

Due to their antimicrobial activity against important human and animal pathogens, bacteriocins are interesting alternatives for conventional antimicrobials in a number of applications (Cotter et al., 2013; Meade et al., 2020; Soltani et al., 2021). However, their therapeutic application and market entry of new bacteriocins are hampered by a lack in easy and cost-effective production and purification at industrially relevant titers (Abbasiliasi et al., 2017; Johnson et al., 2017). Currently processes for industrial scale production of bacteriocins rely on natural producer strains grown on complex media often supplemented with milk or whey (de Arauz et al., 2009; Abbasiliasi et al., 2017), which requires expensive downstream processing and/or results in low product purity. Also, some bacteriocins are naturally produced by pathogenic bacteria that are not suitable for biotechnological production (EFSA Panel on Biological Hazards et al., 2022). One possibility that addresses these obstacles is transfer of production of bacteriocins to recombinant strains of industrial workhorse organisms.

In recent years, we explored the potential of the Gram-positive diderm bacterium *Corynebacterium glutamicum* as a host for recombinant bacteriocin production (Goldbeck et al., 2021; Desiderato et al., 2022; Weixler et al., 2022; Christmann et al., 2023). Due to its long history as a biotechnological platform organism, many genetic tools, cheap and defined minimal media and industrial-scale processes are established (Becker et al., 2018; Wolf et al., 2021; Lee and Jeong, 2022). *C. glutamicum* has several advantages for recombinant bacteriocin production, e.g., the GRAS (generally regarded as safe) status, low extracellular protease activity (Vertès, 2013) and functional protein secretion pathways (Freudl, 2018). Secretion of various recombinant proteins via the Sec pathway of *C. glutamicum* was successfully demonstrated in the mg L^–1^ to g L^–1^ scale (Lee and Jeong, 2022).

The linear and non-modified bacteriocin garvicin Q (GarQ) is a class IId peptide naturally produced by *Lactococcus garvieae* and *Lactococcus petauri* strains (Tosukhowong et al., 2012; Desiderato et al., 2022). The peptide has activity against a wide range of Gram-positive bacteria including *Listeria* spp., *Lactococcus* spp. and *Enterococcus* spp. (Tymoszewska et al., 2017). Similar to the class IIa bacteriocin pediocin PA-1, GarQ uses the transmembrane subunits IIC and IID of the mannose phosphotransferase system (PTS*^Man^*) as receptor to exert its activity (Tymoszewska et al., 2017). Sensitive bacteria are then killed by disruption of membrane integrity and intracellular pH homeostasis (Desiderato et al., 2022). The ABC-transporter GarC and likely the accessory protein GarD are responsible for processing and secretion of GarQ via an N-terminal SP containing the conserved double-glycine-motif (Tosukhowong et al., 2012; Desiderato et al., 2022). Recently established recombinant production of GarQ in *C. glutamicum* using GarCD (Desiderato et al., 2022) but this recombinant strain produced about 4-fold lower levels of GarQ compared to the natural producer *L. petauri* B1726 (Desiderato et al., 2022).

The cell envelope of *L. petauri* B1726 has the typical topology of Gram-positive LAB with a cytoplasmic membrane and a thick peptidoglycan sacculus (Chapot-Chartier and Kulakauskas, 2014). Successful expression of membrane proteins such as GarCD depends on several factors such as membrane lipid composition and can be challenging or even impossible if the intended heterologous host has a cell envelope architecture that differs from the native host (Opekarová and Tanner, 2003). Corynebacteria are diderm bacteria that also stain Gram-positive but have an unusual, complex cell envelope consisting of a cytoplasmic membrane, a peptidoglycan layer that is linked to a arabinogalactan polymer and an outer membrane of mycolic acids similar to outer membrane of Gram-negative bacteria (Burkovski, 2013). Thus, wrong insertion into the cell envelope or compromised functionality of GarCD in *C. glutamicum* may constitute a bottleneck for recombinant production of GarQ by *C. glutamicum*.

In the present study, we established and improve production of GarQ by *C. glutamicum* via the Sec-pathway because we consider GarQ and interesting and suitable bacteriocin for heterologous production using this biotechnological workhorse organism for several reasons. First of all, natural GarQ producers *L. garvieae* and *L. petauri* are biosafety level 2 pathogens hampering large-scale production of GarQ with these organisms. Second, *C. glutamicum* lacks a PTS*^Man^*. Thus, *C. glutamicum* is not sensitive to GarQ, which is a prerequisite for the production at high titers. Furthermore, GarQ is a linear peptide without any posttranslational modifications and therefore an ideal candidate for secretion via the Sec-pathway. Release of GarQ into culture supernatants was improved by minimizing adsorption of the peptide to the producer cells. GarQ titers in supernatants were further improved by using a HtrA-deficient strain and conditions of low oxygenation. The optimized production strategy resulted in stable production of active GarQ at titers above 100 μg ml^–1^.

## 2 Materials and methods

### 2.1 Strains and growth conditions

*Corynebacterium glutamicum* was routinely cultivated at 30°C and under shaking conditions (130 rpm). For growth experiments, *C. glutamicum* strains were cultivated overnight (O/N) in 2xTY medium. Bacteria of these O/N cultures were washed with sterile 0.9% (w/v) NaCl and used to inoculate a modified CGXII minimal medium without urea containing glucose at 2% (w/v) as carbon source (CGXII-U) (Desiderato et al., 2022) to an optical density (OD_600_) of ∼2. For optimization of recombinant GarQ production, 0.5 g L^–1^ CaCl_2_ and/or 0.5% (v/v) Tween 80 were supplemented as indicated. These media will be referred to as CGXII-U+C (CGXII-U with CaCl_2_), CGXII-U+T (CGXII-U with Tween 80), and CGXII-U+CT (CGXII-U with CaCl_2_ and Tween 80). *Lactococcus lactis* IL1403/pNZ-pHin2*^Lm^* was cultivated in M17 medium with 0.5% (w/v) glucose at 30°C with aeration on an orbital shaker. *Listeria monocytogenes* EGDe/pNZ-pHin2*^Lm^* was cultivated in BHI medium at 37°C with aeration on an orbital shaker. For GarQ production by the native producer, *L. petauri* B1726 was cultivated statically in M17 medium supplemented with 2% (w/v) glucose at 30°C overnight in Schott glass bottles. Media were supplemented with 25 μg kanamycin ml^–1^ (*C. glutamicum*), 10 or 7.5 μg ml^–1^ chloramphenicol (*L. lactis* or *C. glutamicum*) and/or 0.2 mM IPTG (*C. glutamicum*) as appropriate. All strains are listed in Table 1.

**TABLE 1:** Bacterial strains used in the present study.

Species/strain	Relevant characteristics	Source/References
***Lactococcus petauri***		
B1726	Natural garvicin Q producer, isolated from fermented balsam pear	Desiderato et al., 2022
***Lactococcus lactis***		
IL1403/pNZ-pHin2*^Lm^*	Biosensor for bacteriocin activity by expression of pHluorin2, derivative of strain IL1403	Desiderato et al., 2022
***Listeria monocytogenes***		
EGDe/pNZ-pHin2*^Lm^*	Biosensor for bacteriocin activity, expression of pHluorin2, derivative of serotype 1/2a type strain EGDe	Reich et al., 2022
***Escherichia coli***		
DH5α	Cloning host	Hanahan, 1983
***Corynebacterium glutamicum***		
CR099	ATCC13032 derivative; cured of prophages CGP1, CGP2, and CGP3 and of insertion elements ISCg1 and ISCg2	Baumgart et al., 2013
CR099/pCMEx8-SP_*XYZ*_-*garQ*	CR099 harboring plasmid pCMEx8 with different Sec-dependent signal peptides (SP) GarQ fusions.	This study
CR099/pXMJ19	CR099 harboring the empty expression vector pXMJ19	This study
CR099/pXMJ19-SP*_*ywaD*_-garQ-*v1	CR099 harboring pXMJ19-SP*_*ywaD*_-garQ-*v1 with linker (2 amino acid residues) between SP*_*ywaD*_* and GarQ coding sequence	This study
CR099/pXMJ19-SP*_*ywaD*_-garQ-*v2	CR099 harboring a derivative of pXMJ19-SP*_*ywaD*_-garQ-*v1, linker (2 amino acid residues) between SP*_*ywaD*_* and GarQ coding sequence deleted	This study
CR099/pPBEx2-*garQICD^Cgl^*	CR099 harboring the plasmid pPBEx2-*garQICD^Cgl^* for IPTG-inducible expression of GarQ biosynthesis genes codon-optimized for *C. glutamicum*	Desiderato et al., 2022
ATCC13032	Wild type strain	ATCC^a^
K9	HtrA protease biosensor strain; *htrA*::*htrA*’-*eypf* (replacement of *htrA* by *htrA*’-*eyfp*)	Jurischka et al., 2020
K9/pXMJ19- SP*_*ywaD*_-garQ-*v2	ATCC13032 K9 harboring pXMJ19-SP*_*ywaD*_-garQ-*v2	This study
K9/pXMJ19- SP*_*ywaD*_-garQ*^M5F^	ATCC13032 K9 harboring pXMJ19-SP*_*ywaD*_-garQ*^M5F^, linker (2 amino acid residues) between SP*_*ywaD*_* and GarQ^M5F^ coding sequence deleted	This study

^*a*^ATCC, American type culture collection.

### 2.2 Molecular biology procedures

Cloning procedures were performed using standard reagents following instructions of the manufacturers. Primers were purchased from a commercial service provider (Eurofins Genomics, Germany) and sequences are listed in Supplementary Table 1. For construction of the Sec-SP library (plasmids pCMEx8-SP_*XYZ*_-*garQ*), an automated protocol for plasmid construction using an OT-2 robot (Opentrons, USA) was applied as previously described (Müller et al., 2022). Briefly, the *garQ* gene lacking the nucleotide sequence for its native SP was amplified from pPBEx2-*garQICD^Cgl^* and inserted into pCMEx8-[SP_*XYZ*_] by Golden Gate Assembly using the type II restriction enzyme *Bsa*I (New England Biolabs). Restriction digestion and capillary electrophoresis were used to check for positive clones. For construction of pXMJ19-SP*_*ywaD*_-garQ*-v1, the gene coding for a fusion peptide consisting of the Sec-dependent SP of YwaD of *B. subtilis* and GarQ was amplified together with its ribosomal binding site from pCMEx8-SP*_*ywa*_-garQ* and new restriction sites were introduced by primers with appropriate adapters containing *Pst*I and *BamH*I restriction sites. The PCR fragment was digested with these enzymes and ligated into linearized pXMJ19. For construction of pXMJ19-SP*_*ywaD*_-garQ*-v2, SP*_*ywaD*_* and *garQ* were separately amplified from pXMJ19-SP*_*ywaD*_-garQ*-v1 lacking the codons for the two amino acid linker between SP and GarQ. The two fragments were fused by overlapping extension PCR and sub-cloned into pJET1.2 (ThermoFisher, Germany) by blunt-end ligation. Then SP*_*ywaD*_-garQ*-v2 was excised from the sub-cloning vector using restriction by *Xba*I and *Pst*I and ligated into linearized pXMJ19. For construction of pXMJ19-SP*_*ywaD*_-garQ^M5F^*, a SP*_*ywaD*_-garQ^M5F^* gene with the mutations for the M5F amino acid exchange was synthesized by a commercial service provider (Eurofins Genomics) and inserted into *Xba*I/*Pst*I-linearized pXMJ19. All constructs were verified by Sanger sequencing by a commercial service provider (Microsynth Seqlab, Germany).

### 2.3 pHluorin2 assay

The pHluorin2 assay was performed according to a previously published protocol using reporter strains *L. lactis* IL1403/pNZ-pHin2*^Lm^* or *L. monocytogenes* EGDe/pNZ-pHin2*^Lm^* (Desiderato et al., 2022; Reich et al., 2022) and Listeria minimal buffer (LMB) with slight modifications. Instead of 100 mM 3-(*N*-morpholino)propanesulfonic acid (MOPS) as a buffering agent, 200 mM 2-(*N*-morpholino)ethanesulfonic acid were used. The assay is based on the two distinct excitation peaks of pHluorin2 at 400 and 480 nm. Fluorescence intensity (FI) at these two excitation peaks shifts in response to changes in pH in a ratiometric manner: with increasing pH, FI after excitation at 400 nm increases and FI after excitation at 480 nm decreases. Under steady-state conditions, biosensor bacteria expressing pHluorin2 are able to maintain an intracellular pH (pH_*i*_) of 7.6–8.0 even in LMB with a pH of 6.2. However, when exposed to compounds that disrupt membrane integrity, pH_*i*_ will rapidly change to the pH of LMB, and this shift can be detected by the changes in FIs after excitation at 400 and 480 nm.

For quantification of GarQ activities, standards with defined concentration (range 0.03–2.0 μg ml^–1^) of chemically synthesized peptides were used to establish a calibration curve by plotting ratios of pHluorin2 fluorescence intensities at 520 nm after excitation at 400 and 480 nm (ratio RFU 400/480) against peptide concentration. The equation of the calibration curve was calculated as a nonlinear regression using a semilogarithmic fit with least square fitting. The coefficient of determination R^2^ was > 0.96 in all cases. These calibration curves were used to calculate mass concentration of GarQ in unknown samples based on measurements of ratio RFU 400/480. Statistical analysis was performed by ANOVA with the Bonferroni post-test to calculate *p*-values adjusted for multiple comparisons. GraphPad Prism 6 software was sued to compute nonlinear regression and ANOVA. Chemically synthesized GarQ was obtained from the Functional Peptidomics core facility of Ulm University. Dried, synthetic GarQ had a purity of > 95% and was dissolved in HPLC water for further use.

### 2.4 Purification and identification of GarQ

GarQ was purified from culture supernatants of *C. glutamicum* by sequential hydrophobic interaction chromatography (HIC) and reverse-phase chromatography (RPC) as previously described (Desiderato et al., 2022). After HIC, GarQ was eluted with HPLC-grade water. Elution buffer in RPC was 80% acetonitrile and 0.05% TFA in water and GarQ eluted at 30% elution buffer.

Mass spectrometry analysis of purified GarQ peptide fractions was performed based on a previously described method (Desiderato et al., 2022). Lyophilized peptide fractions were concentrated to 1 mg ml^–1^. Aliquots of peptide solutions were subjected to tryptic digestion, with 1 μg trypsin in a total volume of 100 μl for 5 h at 42°C as recommended by the supplier. Solutions of intact and digested peptides were diluted 1:2 with LC-MS-grade H_2_O prior to Liquid chromatography mass spectrometry (LC-MS).

Liquid chromatography mass spectrometry was conducted with an Agilent 1260 Infinity system (Agilent Technologies, Waldbronn, Germany) coupled to a quadrupole time-of-flight mass spectrometer (TripleTOF6600, AB Sciex, Darmstadt, Germany). LC was performed with an Ascentis^®^ Express Peptide ES-C18, 2.7 μm HPLC column (53307-U, Merck, Darmstadt, Germany) with a flow rate of 200 μL min^–1^ and the mobile phases (A) 0.1% formic acid in water and (B) acetonitrile. The elution gradient was as follows: 0 min, 3% B; 70 min, 40% B; 78 min 40% B, 79 min 60% B, 89 min 60% B, 90 min 3% B followed by a 12 min equilibration time between injections. Column temperature was set to 21 °C and injection volume to 10 μL. MS was conducted with a TurboV ion source operated in positive ionization mode. Ion spray voltage was set to 5.5 kV, source temperature to 450 °C, curtain gas to 35 psi, and the support gases GS1/GS2 to 50 psi/50 psi. All gases were nitrogen.

For analysis of undigested peptide, the quadrupole time-of-flight (QToF) mass spectrometer was operated in ToF mode with a dwell time of 250 ms and for digested peptide, the QToF mass spectrometer was operated in data-dependent acquisition (DDA) mode. Based on a ToF survey scan with a dwell time of 250 ms, product ion scans with a dwell-time of 100 ms were automatically performed for a maximum of 40 ions with a mass-to-charge ratio > 300 m/z and a charge of 2 to 4 passing an intensity threshold of 150 cps. After acquisition, mass-to-charge ratios were excluded from the potential candidate-ion list for 12 s. Declustering potential was set to 120 V, collision energy spread to 5 V and mass tolerance to 25 ppm.

Acquired mass spectra were analyzed with PeakView 2.1 (AB Sciex, Darmstadt, Germany). Protein identification was performed with the ProteinPilot 5.1 software (AB Sciex, Darmstadt, Germany).

### 2.5 Adsorption assay

The adsorption assays were performed as previously described (Desiderato et al., 2022) with slight modifications. Overnight cultures of *C. glutamicum* were washed three times with phosphate-buffered saline. The universal buffer UB3 (20 mM HEPES, 20 mM Bis-Tris and 20 mM sodium acetate) was used to allow adsorption studies at different pH values without changing the buffer composition (Brooke et al., 2015). Bacteria were resuspended at an OD_600_ of 3 in UB3 buffer set to different pH containing HIC purified GarQ. After incubation of the peptide with bacteria for 1 h at 30°C, bacteria were pelleted by centrifugation (11,000 × *g*, 5 min, room temperature) and the adsorption supernatant (ASN) was collected. In parallel, GarQ was incubated at the same conditions and buffer composition without bacteria as a control. Antimicrobial activity of two-fold serial dilution of ASN and control samples was then determined in growth inhibition assay using *L. lactis* IL1403 as indicator strain as described previously (Desiderato et al., 2022). To calculate bacteriocin activity, a nonlinear fit based on the Gompertz function was calculated using GraphPad Prism 6 software. The coefficient of determination R^2^ of the Gompertz function was above 0.97 in all cases, indicating that the function is suitable. Arbitrary units (AU) of bacteriocin activity of sample was defined as the X-value of the inflection point (1/K) of the Gompertz function. The percentage of adsorbed GarQ was calculated according to following formula:

$$\mathrm{adsorption}\left[\%\right]=100-(\frac{\mathrm{bacteriocin}\,\mathrm{activity}\,\mathrm{in}\,\mathrm{ASN}}{\mathrm{bacteriocin}\,\mathrm{activity}\,\mathrm{in}\,\mathrm{original}\,\mathrm{sample}}\times 100)$$

### 2.6 Flow cytometry analysis

*Corynebacterium glutamicum* strains were cultivated in CGXII-U or normal CGXII minimal medium (with urea). Samples were taken at the end of exponential phase and in stationary phase. Cells were diluted 1:100 in PBS and 50 μl of cell suspension was analyzed for fluorescence intensity of the eYFP reporter (FI eYFP) of *C. glutamicum* K9 strains in an Amnis^®^ CellStream^®^ device (Luminex) equipped with a 488 nm laser (emission wavelength 528 nm). The flow speed was set to “slow.” The laser powers were: 15% for forward scatter (FSC) and side scatter (SSC) and 40% for the 488 nm laser (excitation of eYFP). Bacteria were gated based on FSC and SSC and FI eYFP was recorded for at least 5,000 bacteria per sample. Flow cytometry data was analyzed using the FlowJo analysis software (v10).

### 2.7 Proteomic analysis

For proteomic analysis, *C. glutamicum* ATCC13032 was cultivated in a four-fold parallel bioreactor setup (DASGIP^®^ Parallel Bioreactor System, Eppendorf SE, Germany) with a filling volume of 1 L each. Bacteria were grown in CGXII-U medium without MOPS and pH was maintained at defined levels (pH = 5 or 8) using 4 M KOH or 6 M H_3_PO_4_. Ample oxygen supply was guaranteed by using an oxygen controller set to 30% dissolved oxygen and using a cascade of stirrer speed, gas flow and oxygen concentration in inlet air. The bioreactors were inoculated to an OD_600_ of 1.5. Samples for proteome analysis were taken in exponential and stationary growth phase in technical triplicates. For each replicate, 1.6 ml culture broth were samples to a proteome vial and centrifuged for 5 min at 4°C and 21,500 × *g*. The supernatant was discarded, and pellets were frozen in liquid nitrogen and stored at −80°C. Proteomic analysis was carried out as described elsewhere (Unrean et al., 2018). The mass spectrometry proteomics data have been deposited to the ProteomeXchange Consortium via the PRIDE (Perez-Riverol et al., 2022) partner repository with the dataset identifier PXD046499.

## 3 Results

### 3.1 Identification of a suitable Sec-dependent secretion signal

**FIGURE 1:**
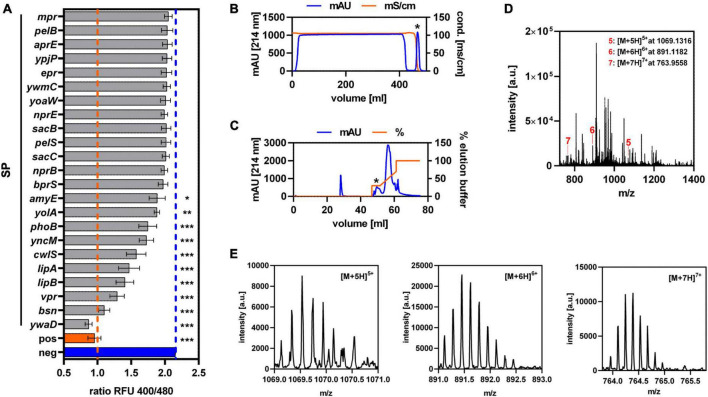
Establishment of Sec-dependent secretion of GarQ by *C. glutamicum*. **(A)** Screening of 23 different Sec-dependent SPs of *Bacillus subtilis* for secretion of GarQ by pHluorin2 assay using *L. lactis* IL1403/pNZ-pHin2*^Lm^* as biosensor. Values are ratios of fluorescence intensity (emission at 520 nm) of the biosensor after excitation at 400 and 480 nm (ratio RFU 400/480) and are mean ± standard deviation (SD) of three independent experiments (i.e., supernatants of independent cultivations of the strains of the SP library). As controls, biosensors treated with purified GarQ (pos) or buffer only (neg) were used. Statistical analysis was performed by ANOVA with the Bonferroni post-test to calculate *p*-values adjusted for multiple comparisons and untreated biosensors (i.e., neg) set as the control condition (*p < 0.05; **p < 0.01; ***p < 0.001). **(B,C)** Purification of GarQ from supernatants of *C. glutamicum* CR099/pXMJ19-SP*_*ywaD*_*-*garQ*-v2 by hydrophobic interaction chromatography **(B)** and reverse-phase chromatography **(C)**. Peaks containing antimicrobial activity as indicated by pHluorin2 assays (marked by *) were selected for further purification and mass spectrometry. **(D)** LC–MS analysis of combined RPC elution fraction harboring antimicrobial activity. The indicated peaks (red lines and numbers) with m/z ratios corresponding to the calculated monoisotopic mass of GarQ (5340.63 Da) carrying five (5: [M+5H]^5+^, m/*z* = 1069.1316), six (6: [M+6H]^6+^, m/*z* = 891.1182), or seven (7: [M+7H]^7+^, m/*z* = 763.9558) positive charges. **(E)** Zoom on the peaks of the [M+5H]^5+^ (m/z spacing: 0.200), [M+6H]^6+^ (m/z spacing: 0.167), and [M+7H]^7+^ (m/z spacing: 0.143) ions indicated in panel **(D)**. In **(B–E)** results of one representative of at least three independent experiments are shown.

To establish Sec-dependent secretion of GarQ, an existing library of known Sec-dependent SPs of *Bacillus subtilis* was used to replace the native double-glycine-type signal sequence by automized plasmid construction (Müller et al., 2022). This yielded constructs of 23 different N-terminal SPs fused to the GarQ-coding sequence. Following transformation into *C. glutamicum*, all strains were cultivated in CGXII-U medium and GarQ activity was determined in culture supernatants after 24 h of cultivation by pHluorin2 assay (Figure 1A). A significant response of the sensor *L. lactis* IL1403/pNZ-pHin2*^Lm^* was detected with supernatants of a total of 10 strains of the library. Highest activity and thus most efficient secretion was observed with the SP of the *ywaD* gene (SP*_*ywaD*_*) and the corresponding *SP_*ywaD*_-garQ* fusion was selected for further experiments.

As a remnant of the cloning procedure, GarQ derivatives expressed using the constructs of the SP library contain a linker of two amino acid residues (glutamate and phenylalanine) at the N-terminus of GarQ following cleavage by the signal peptidase. To obtain GarQ by Sec-dependent secretion without these two amino acids, we generated a second version of our construct. Both strains, *C. glutamicum* CR099/pXMJ19-SP*_*ywaD*_-garQ*-v1 (with linker) and *C. glutamicum* CR099/pXMJ19-SP*_*ywaD*_-garQ*-v2 (without linker), showed comparable growth characteristics and had comparable levels of GarQ activity in their supernatants (Supplementary Figure 1). This indicates that presence/absence of the linker had no effect on activity and/or secretion. To ensure that GarQ is secreted and processed properly by the Sec translocon, the active compound was purified from culture supernatants of *C. glutamicum* CR099/pXMJ19-SP*_*ywaD*_-garQ*-v2 by sequential HIC and RPC (Figures 1B, C). Three distinct peaks with mass/charge ratios (m/z) of 1069.1316, 891.1182, or 763.9558, respectively, were identified by LC-MS of pooled RPC fractions containing antimicrobial activity (Figures 1D, E). These peaks correspond to m/z of the five-, six and seven-fold protonated ions of GarQ and are in good agreement with the calculated monoisotopic mass of natural GarQ (5340.63 Da). Trypsin digestion and subsequent mass spectrometry analysis further confirmed the presence and correct processing of GarQ in culture supernatants of the recombinant *C. glutamicum* strain (data not shown).

For better estimation of mass concentrations of GarQ, we established calibration curves for quantification of GarQ by the pHluorin2 assay using standards of chemically synthesized GarQ of known concentration serially diluted in CGXII-U medium (Supplementary Figure 2). Using this approach, we determined that *C. glutamicum* CR099/pXMJ19-SP*_*ywaD*_-garQ*-v2 produces 7.2 ± 1.5 mg L^–1^ GarQ after cultivation in CGXII-U medium for 24 h. This is about 3.5-fold higher than levels observed with the previously published GarQ production strain *C. glutamicum* CR099/pPBEx2-*garQICD^Cgl^* (Desiderato et al., 2022) under the same conditions (2.0 ± 0.3 mg L^–1^). However, it is still by far lower than GarQ levels in supernatants of the native producer *L. petauri* B1726 grown in GM17 for 24 h (198.6 ± 5.6 mg L^–1^).

### 3.2 Extracellular pH affects adsorption of GarQ to *C. glutamicum*

Production of bacteriocins by their native producers is affected by pH and generally LAB produce higher titers in acidic conditions (Abbasiliasi et al., 2017). Likewise, we previously reported that acidic pH enhances recombinant production of pediocin PA-1 and GarQ by *C. glutamicum* (Desiderato et al., 2022; Christmann et al., 2023). Also, GarQ is strongly adsorbed by *C. glutamicum* and other bacterial cells (Desiderato et al., 2022) at neutral pH and we have made similar observations for other bacteriocins including nisin Z and pediocin PA-1 (data not shown). To better understand the adsorption of GarQ by *C. glutamicum* cells, we tested the impact of different pH values (4, 5, 6, 7, and 8) on adsorption (Table 2; Supplementary Figure 3).

**TABLE 2:** Effect of pH on GarQ activity and adsorption to *C. glutamicum* CR099.

pH	GarQ activity (AU)	ASN activity (AU)	Adsorption (%)
4	1,450	1,183	18
5	1,566	535	66
6	1,347	173	87
7	720	68	90
8	494	62	87

Adsorption (%) is calculated by comparing the activity in adsorption supernatants (ASN) of *C. glutamicum* at the indicated pH to the activity of GarQ incubated under the same conditions without bacteria. Data of activity assays is shown in Supplementary Figure 3. Values are mean of three independent experiments.

In general, we observed a decrease in adsorption at lower pH. At pH = 6–8, about 90% of the peptide adsorbed to the cells. At pH = 5 adsorption was reduced to 66% and at pH = 4 only 18% of the peptide were adsorbed by bacteria. Concomitantly, we also observed that activity of GarQ was reduced at pH = 7–8.

### 3.3 CaCl_2_ and Tween 80 reduce adsorption of GarQ and increases GarQ levels in supernatants

To reduce adsorption of GarQ to biomass of *C. glutamicum* and improve release into the supernatant, we sought to modify cultivation conditions. In previous studies, CaCl_2_ was shown to improve the heterologous production of secreted proteins with *C. glutamicum* (Teramoto et al., 2011; Freier et al., 2016), to improve resistance of *C. glutamicum* to nisin, and to increase recombinant pediocin PA-1 production (Weixler et al., 2022; Christmann et al., 2023). Tween 80 is a non-ionic detergent that is used as a solubilizer for proteins and as emulsifying agent in food (Linke, 2009; Duquesne and Sturgis, 2010). Thus, we tested the effects of CaCl_2_ and Tween 80 on adsorption of GarQ to *C. glutamicum* (Table 3; Supplementary Figure 4).

**TABLE 3:** Effect of CaCl_2_ and Tween 80 on the adsorption of GarQ to *C. glutamicum*.

UB3, pH = 7		GarQ activity (AU)	ASN activity (AU)	Adsorption (%)
CaCl_2_ (g L^–1^)	Tween 80 [% (w/v)]			
–	–	594	90	85
0.5	–	773	154	80
1	–	942	194	79
2	–	1,068	238	78
–	0.1	884	190	79
–	0.5	898	324	64
–	1	1,002	317	68
0.5	0.5	1,274	1,135	11

Adsorption (%) is calculated by comparing the activity in adsorption supernatants (ASN) of *C. glutamicum* in UB3 at pH = 7 with the indicated supplements to the activity of GarQ incubated under the same conditions without bacteria. Data of activity assays is shown in Supplementary Figure 4. Values are mean of three independent experiments.

The effects of Tween 80 and CaCl_2_ individually were rather moderate. Supplementation with 0.5–2 g L^–1^ CaCl_2_ reduced adsorption to ∼80% and the addition of 0.5% (w/v) Tween 80 reduced adsorption to 64%. Interestingly, a combination of 0.5 g L^–1^ CaCl_2_ and 0.5% (w/v) Tween 80 strongly reduced adsorption of GarQ to 11%, suggesting a synergistic effect.

**FIGURE 2:**
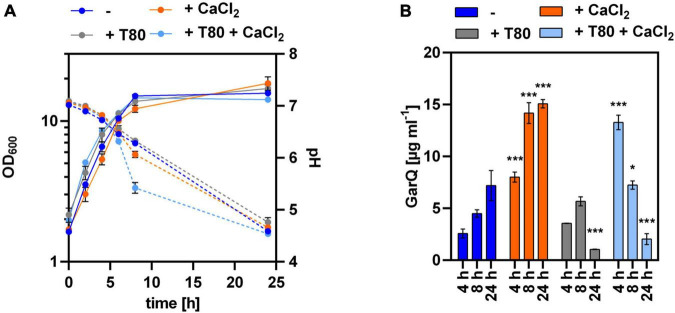
Impact of Tween 80 and CaCl_2_ supplementation on recombinant GarQ production by *C. glutamicum*. **(A)** Biomass (OD_600_; solid lines) and pH (broken lines) in supernatants of *C. glutamicum* CR099/pXMJ19-SP*_*ywaD*_*-*garQ*-v2 during cultivation in CGXII-U (dark blue; -), CGXII-U+C (orange; +CaCl_2_), CGXII-U+T (gray, T80), or CGXII-U+CT (light blue, T80+CaCl_2_). **(B)** Concentrations of GarQ in supernatants of these cultures at the indicated timepoints were determined by pHluorin2 assays using *L. lactis* IL1403/pNZ-pHin2*^Lm^* as biosensor. Mass concentrations were calculated using a calibration curve established with standards of synthetic GarQ. All values are mean ± SD of *n* = 3 independent cultivations per condition. Statistical analysis was performed comparing levels of GarQ between conditions at each timepoint by ANOVA and Bonferroni’s post-test to calculate *p*-values adjusted for multiple comparisons with cultures in CGXII-U (–) set as control condition (*p < 0.05; ***p < 0.001).

Based on these results, we cultivated *C. glutamicum* CR099/pXMJ19-*ywaDSPgarQ*-v2 in CGXII-U, CGXII-U+C (containing 0.5 g L^–1^ CaCl_2_), CGXII-U+T [with 0.5% (w/v) Tween 80], or CGXII-U+CT (containing both supplements) and compared GarQ levels in supernatants (Figure 2). Supplementation with CaCl_2_ alone increased GarQ titers at all timepoints analyzed and a maximum of 15.1 ± 0.4 mg L^–1^ GarQ was observed after 24 h. When only Tween 80 was supplemented, the GarQ production in exponential phase was comparable to that CGXII-U without supplements (2.6 ± 0.4 mg L^–1^) at *t* = 4 h but decreased in stationary phase. In CGXII-U+CT, we observed increased GarQ levels after 4 h of cultivation (13.3 ± 0.7 mg L^–1^) but the activity decreased drastically to 2.0 ± 0.5 mg L^–1^ after 24 h.

### 3.4 A *C. glutamicum* strain lacking HtrA protease shows increased GarQ production

**FIGURE 3:**
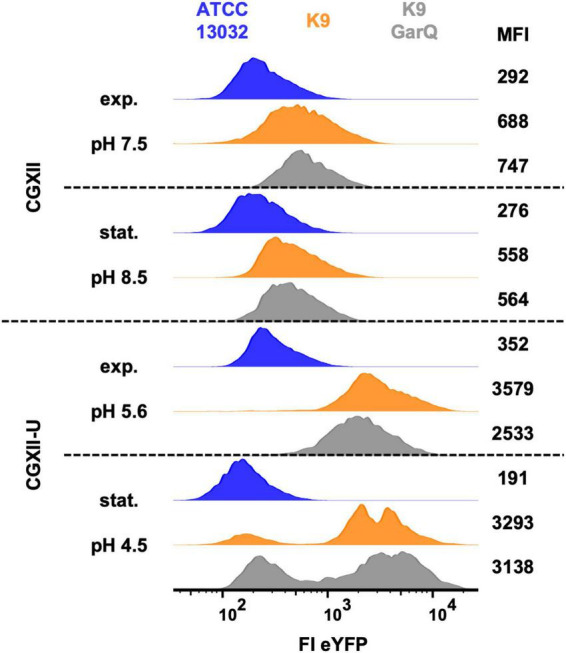
Analysis of *C. glutamicum* ATCC13032 (blue), K9 (orange), and K9/pXMJ19-SP*_*ywaD*_*-*garQ*-v2 (K9-GarQ, gray) for *htrA*-reporter activity. All strains were cultivated in CGXII (upper panels) or CGXII-U (lower panels). Samples for flow cytometry were taken in exponential (exp.) or stationary (stat.) growth phase, pH in supernatants was measured and single bacteria were analyzed for eYFP fluorescence intensity (FI-eYFP) by flow cytometry. Shown are histogram plots of FI eYFP of at least *n* = 5,000 bacterial cells per condition with mean fluorescence intensity (MFI). Results of one representative of at least two independent experiments per condition are shown.

*Corynebacterium glutamicum* is generally believed to have little to no extracellular protease activity (Vertès, 2013). However, the dramatic decrease in GarQ activity in supernatants of *C. glutamicum* CR099/pXMJ19-*ywaDSPgarQ*-v2 grown in CGXII-U+CT at later timepoints of the cultivation prompted us to reconsider proteolytic degradation as a potential problem under these conditions. For other bacteria, it is described that high level heterologous expression of secreted proteins and presence of bacteriocins induces cell envelop/secretion stress and expression of proteases for cleavage of unfolded or misfolded proteins (Jordan et al., 2008; Neef et al., 2020). A protease previously associated with Sec-dependent secretion stress in *C. glutamicum* is HtrA (Jurischka et al., 2020). This was reported for *C. glutamicum* K9, a biosensor that reports secretion stress by expression of the yellow fluorescent protein eYFP from a gene cassette replacing the chromosomal *htrA* gene. To investigate whether HtrA may play a role in reducing GarQ levels, we performed similar experiments under the conditions of GarQ production. *C. glutamicum* K9 was transformed with pXMJ19-SP*_*ywaD–*_garQ*-v2 and the obtained strain as well as its two parental strains K9 and ATCC13032 were grown in standard CGXII or CGXII-U, and eYFP fluorescence was analyzed by flow cytometry (Figure 3).

In normal CGXII medium, pH was slightly increased to 8.5 in stationary growth phase and the two K9 strains showed only marginally increased eYFP fluorescence (approx. 2-fold higher mean fluorescence intensity; MFI) compared to the negative control, i.e., wildtype strain ATCC13032. As observed previously, pH in cultures grown in CGXII-U dropped markedly to 5.6 in exponential and to 4.5 in stationary growth phase. At the same time, eYFP expression of the two K9 strains was markedly increased. Compared to the wildtype strain ATCC13032, MFI of *C. glutamicum* K9 was increased approx. 10-fold and for *C. glutamicum* K9/pXMJ19-SP*_*ywaD*_*-*garQ*-v2 a 7-fold increase in eYFP fluorescence was observed. Although for a subpopulation of these strains eYFP fluorescence returned to baseline in stationary growth phase, still a considerable fraction remains eYFP positive. No clear difference was observed between *C. glutamicum* K9 and K9/pXMJ19-SP*_*ywaD–*_garQ*-v2. These results indicate that HtrA expression is increased in acidic environments but is not affected by expression/secretion of GarQ.

Additionally, LC-MS proteome analysis of *C. glutamicum* ATCC13032 cultivated in bioreactors in CGXII-U without MOPS at defined pH values confirmed that HtrA levels are increased at acidic extracellular pH. At pH = 5, signals for HtrA protein were increased 7.6-fold (*p* = 2.09E-08) and 2.4-fold (*p* = 0.01) in exponential and stationary growth phase, respectively, compared to pH = 8.

**FIGURE 4:**
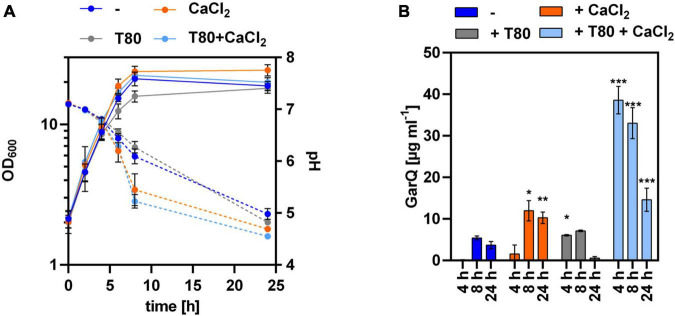
Lack of HtrA protease increases GarQ levels produced by *C. glutamicum*. **(A)** Biomass (OD_600_; solid lines) and pH (broken lines) in supernatants of *C. glutamicum* K9/pXMJ19-SP*_*ywaD*_*-*garQ* during cultivation in CGXII-U (dark blue; -), CGXII-U+C (orange; +CaCl_2_), CGXII-U+T (gray, T80), or CGXII-U+CT (light blue, T80+CaCl_2_). **(B)** Concentrations of GarQ in supernatants of these cultures at the indicated timepoints were determined by pHluorin2 assays using *L. lactis* IL1403/pNZ-pHin2*^Lm^* as biosensor. Mass concentrations were calculated using a calibration curve established with standards of synthetic GarQ. All values are mean ± SD of *n* = 3 independent cultivations per condition. Statistical analysis was performed comparing levels of GarQ between conditions at each timepoint by ANOVA and Bonferroni’s post-test to calculate *p*-values adjusted for multiple comparisons with cultures in CGXII-U (–) set as control condition (*p < 0.05; **p < 0.01; ***p < 0.001).

Based on these results, we hypothesized that increased expression of *htrA* under the conditions of GarQ production may cause proteolytic degradation of the product and result in decreased activity in supernatants at later timepoints of the fermentation. To test this hypothesis, we analyzed GarQ production by *C. glutamicum* K9/pXMJ19-SP*_*ywaD–*_garQ*-v2 lacking a functional *htrA* gene (Figure 4). When grown in CGXII-U+CT, GarQ titers of 38.6 ± 3.3 mg L^–1^ were observed after 4 h of cultivation. This is a ∼3-fold increase compared to the highest GarQ levels observed with *C. glutamicum* CR099/pXMJ19-SP*_*ywaD–*_garQ*-v2, i.e., the strain harboring an intact *htrA* gene (Figure 2). Also, GarQ concentrations remained high at *t* = 8 h of fermentation. Of note, lack of a functional *htrA* gene had no effect on GarQ levels in CGXII-U, CGXII-U+C or CGXII-U+T. This suggests that HtrA causes proteolytic degradation of GarQ when expressed and deletion of *htrA* improves production only under the optimized conditions, i.e., growth in CGXII-U+CT, i.e., when both CaCl_2_ and Tween 80 are supplemented. Nevertheless, we still observed a decrease in GarQ activities at later timepoints of the fermentation (14.6 ± 2.8 mg L^–1^ GarQ at *t* = 24 h) indicating that there are also other factors that reduce levels of the active product.

### 3.5 Cultivation under low oxygen conditions improves recombinant GarQ production

Recently, we were able to show that fermentation under low oxygen conditions in bioreactor or non-baffled flasks improves production of the class IIa bacteriocin pediocin PA-1 (Goldbeck et al., 2021; Christmann et al., 2023). A possible explanation for this effect is that pediocin PA-1 is (partially) inactivated by oxidation of the methionine residue in position 31 (Bédard et al., 2018). Similar to pediocin PA-1, GarQ contains a methionine residue at position 5 of the active peptide. Hence, inactivation of the peptide by oxidation of this methionine may occur. Indeed, re-evaluation of the LC-MS data of the purified GarQ fraction (Figure 1) revealed that mass spectra also contained signals with of 1072.326 m/z (spacing: 0.200) and 893.772 m/z (spacing: 0.167) corresponding to the five- and six-fold protonated ions of oxidized GarQ (Supplementary Figure 5A). We also observed a loss of GarQ activity by about 50% after overnight incubation at 30°C, 130 rpm compared to samples stored at −20°C (Supplementary Figure 5B).

**FIGURE 5:**
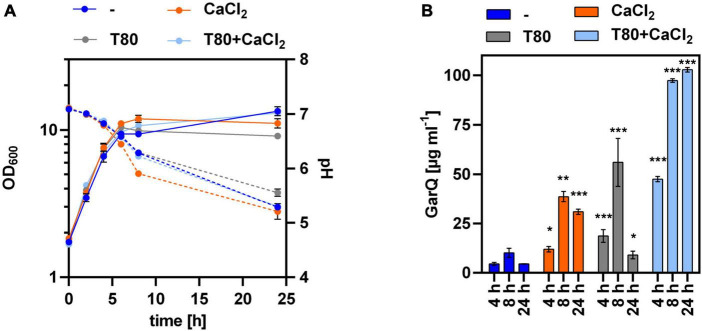
GarQ production by *C. glutamicum* is increased in conditions of reduced aeration. **(A)** Biomass (OD_600_; solid lines) and pH (broken lines) in supernatants of *C. glutamicum* K9/pXMJ19-SP*_*ywaD*_*-*garQ* during cultivation in CGXII-U (dark blue; -), CGXII-U+C (orange; +CaCl_2_), CGXII-U+T (gray, T80), or CGXII-U+CT (light blue, T80+CaCl_2_) in non-baffled flasks. **(B)** Concentrations of GarQ in supernatants of these cultures at the indicated timepoints determined by pHluorin2 assays using *L. lactis* IL1403/pNZ-pHin2*^Lm^* as biosensor. Mass concentrations were calculated using a calibration curve established with standards of synthetic GarQ. All values are mean ± SD of *n* = 3 independent cultivations per condition. Statistical analysis was performed comparing levels of GarQ between conditions at each timepoint by ANOVA and Bonferroni’s post-test to calculate *p*-values adjusted for multiple comparisons with cultures in CGXII-U (–) set as control condition (*p < 0.05; **p < 0.01; ***p < 0.001).

To test if reduced aeration might positively impact on GarQ production, we cultivated *C. glutamicum* K9/pXMJ19-SP*_*ywaD–*_garQ*-v2 in non-baffled shake flasks (Figure 5) and observed a slight increase in GarQ titers to 47.6 ± 1.3 mg L^–1^ in cultures grown in CGXII-U+CT at 4 h of fermentation. However, in contrast to all previous experiments GarQ concentrations further increased toward the end of the fermentation and reached a maximum of 102.8 ± 1.1 mg L^–1^ after 24 h of cultivation. Similarly, increased levels of GarQ were observed in CGXII-U+T at 4 h (18.6 ± 3.2 mg L^–1^) and 8 h (56.0 ± 12.1 mg L^–1^) of cultivation. Of note, reduced aeration had no impact on GarQ levels in CGXII-U or CGXII-U+C.

### 3.6 Activity of a natural GarQ variants

**FIGURE 6:**
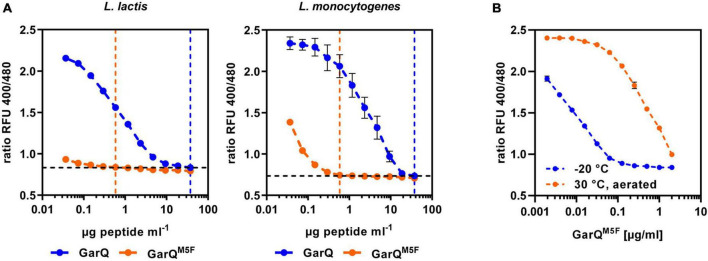
A natural variant of GarQ shows altered properties. **(A)** Activity of GarQ (dark blue) and GarQM5F (orange) or **(B)** GarQ^M5F^ incubated O/N at 30°C with aeration on a rotary shaker (orange) or stored at –20°C (blue) was determined by pHluorin2 assays. *L. lactis* IL1403/pNZ-pHin2*^Lm^* [left panel in **(A,B)**] or *L. monocytogenes* EGDe/pNZ-pHin2*^Lm^* [right panel in **(A)**] were used as sensors. Values are ratios of fluorescence intensity of the biosensor (emission at 520 nm) after excitation at 400 and 480 nm (ratio RFU 400/480) and are mean ± SD of *n* = 3 independent experiments.

Activity of the class II bacteriocin pediocin PA-1 was shown to be sensitive to oxidation of a methionine residue. As GarQ contains a methionine in position 5 of the active peptide, we hypothesized that GarQ may be inactivated by oxidation of this methionine and cultivation in low oxygen conditions may reduce oxidative inactivation Garvicin AG2 is a natural variant of GarQ, in which the methionine in position 5 is changed to a phenylalanine (Maldonado-Barragán et al., 2022). To test if this peptide may be protected against oxidative inactivation, we purchased a synthetic GarQ variant with the M5F amino acid exchange (GarQ*^M5F^*) from a commercial supplier and compared its activity to native GarQ in pHluorin2 assays using *L. lactis* LMG2785/pNZ-pHin*^Lm^* or *L. monocytogenes* EGDe/pNZ-pHin*^Lm^* (Figure 6A).

Surprisingly, GarQ*^M5F^* has markedly higher activity against both biosensors than the native peptide as concentration of GarQ*^M5F^* to achieve a complete shift in fluorescence ratios was 64-fold lower than for GarQ. To test if GarQ*^M5F^* is also protected against oxidative inactivation, we performed activity assays of GarQ*^M5F^* after incubation O/N at 30°C with aeration or stored at −20°C. In contrast to our predictions, activity of GarQ*^M5F^* was reduced by aeration (Figure 6B) and this effect was even more pronounced than oxidative inactivation of the wildtype GarQ (Supplementary Figure 5). This indicates that GarQ*^M5F^* has markedly improved activity, but oxidation of the methionine seems not to be the reason for reduced activity of GarQ after prolonged cultivation or incubation under oxygenated conditions.

**FIGURE 7:**
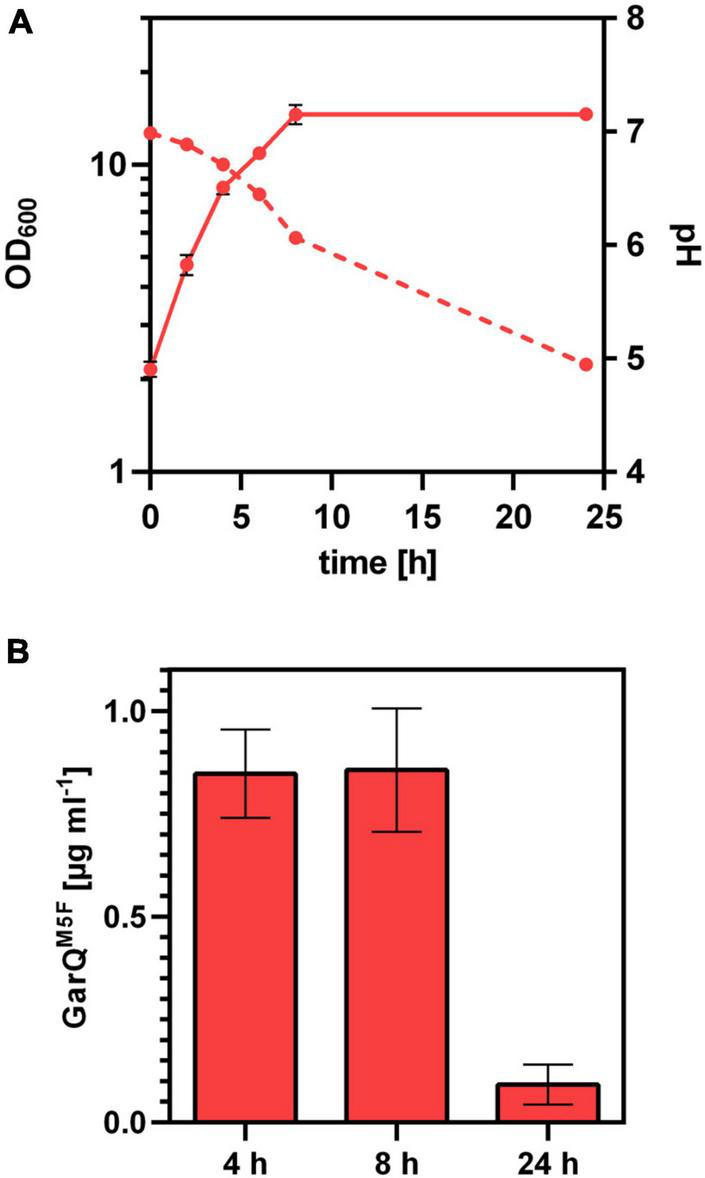
Recombinant production of GarQ^M5F^. **(A)** Biomass (OD_600_; solid lines) und pH (broken lines) in supernatants of *C. glutamicum* K9/pXMJ19-SP*_*ywaD*_*-*garQ*^M5F^ during cultivation in CGXII-U+CT. **(B)** Concentration of GarQ^M5F^ in supernatants of these cultures at the indicated timepoints were determined by pHluorin2 assays using *L. lactis* IL1403/pNZ-pHin2*^Lm^* as biosensor. Mass concentrations were calculated using a calibration curve established with standards of synthetic GarQ^M5F^. All values are mean ± SD of *n* = 3 independent cultivations per condition.

Based on the interesting properties of GarQ*^M5F^*, we generated a *C. glutamicum* production strain again employing Sec-dependent secretion using the YwaD SP. The strain *C. glutamicum* K9/pXMJ19-SP*_ywaD_-garQ^M5F^* was cultivated in CGXII-U+CT in baffled flasks and activities were determined in supernatants at different timepoints during cultivation (Figure 7). Surprisingly, activities were approx. 40-fold lower than with the isogenic strain producing wildtype GarQ cultivated under the same conditions (Figure 4). As observed in all other experiments conducted in baffled flasks, GarQ*^M5F^* activity was decreased by 10-fold at 24 h of incubation again supporting that methionine oxidation is not the reason for loss in activity of GarQ and GarQ*^M5F^* during recombinant production under high oxygen conditions.

## 4 Discussion

Bacteriocins are a heterogeneous group of antimicrobial peptides and proteins produced by bacteria and a few of these peptides are approved and marketed as preservatives in food and beverages and in animal and pet feed (Mills et al., 2011; Chikindas et al., 2018; Silva et al., 2018). As antibiotic resistant variants of a wide range of infectious agents are an increasing concern of human health on a global scale and some bacteriocins are active against these antibiotic resistant pathogens (Ovchinnikov et al., 2016, 2017, 2020), bacteriocins also offer opportunities to combat these infections. However, commercial available bacteriocins are produced exclusively with their natural producers organisms on complex media and are sold as semi-purified preparations or crude fermentates, which limits their use in clinical applications (Abbasiliasi et al., 2017; Johnson et al., 2017; Juturu and Wu, 2018).

To allow easy and cost-effective production and purification at industrially relevant titers, we recently established *C. glutamicum* as a host for recombinant bacteriocin production (Goldbeck et al., 2021; Desiderato et al., 2022; Weixler et al., 2022; Christmann et al., 2023). The approach applied so far is based on expression systems for the prepeptide of a bacteriocin together with the modification and secretion machineries of the native producer. This strategy is essential for bacteriocins containing post-translational modifications such as nisin but might not be required for linear and non-modified peptides. In fact, it was shown for several bacteriocins that the double glycine SPs can be successfully replaced by Sec-dependent SPs (McCormick et al., 1996; Martín et al., 2007; Borrero et al., 2011; Back et al., 2016) and recently a fusion of the SP of lactococcal protein Usp45 and GarQ allowed recombinant GarQ production by *L. lactis* (Feito et al., 2023). To identify a suitable Sec-dependent SP for GarQ secretion by *C. glutamicum* we screened an available library of *B. subtilis* SPs. This library was previously used to screen for secretion of cutinase alone or fused to split-GFP in both *B. subtilis* and *C. glutamicum* (Hemmerich et al., 2016; Müller et al., 2022) and it was shown that efficient expression is affected by differences in G+C content of the coding sequences for SP (from low G+C *B. subtilis*) and the target protein itself (which when derived from *C. glutamicum* has high G+C content). For recombinant expression, the same signal peptide was used for GarQ and the GarQ*^M5F^* variant yet a 40-fold difference in active peptide were observed. Further studies are needed to investigate, why a single amino acid exchange at the N-terminus of the mature bacteriocin close to the SP cleavage site has such a profound impact on secretion efficiency.

Bacteriocins are generally hydrophobic and positively charged peptides and a number of studies show adsorption of bacteriocins to the negatively charged cell envelope of bacteria (Parente et al., 1994; De Vuyst et al., 1996; Callewaert and De Vuyst, 2000). This may constitute a major bottleneck for recombinant bacteriocin production. Indeed, we previously observed that GarQ efficiently adsorbs to various sensitive and insensitive bacteria including *C. glutamicum* (Desiderato et al., 2022). As *C. glutamicum* does not encode the genes for a PTS*^Man^* and is resistant to GarQ (Desiderato et al., 2022), absorption of GarQ is independent of the described receptor and is probably caused by electrostatic interactions of the cationic peptide with the negatively charged cell envelope. In the present study, we confirm and extend these results showing that adsorption of GarQ to *C. glutamicum* is pH-dependent and decreases at acidic pH as observed for several other bacteriocins (Yang et al., 1992). This may explain why acidification of the supernatant during cultivation is needed to obtain measurable GarQ titers with recombinant *C. glutamicum* strains (Desiderato et al., 2022). Similarly, fermentation at acidic pH boosted recombinant production of pediocin PA-1 with *C. glutamicum* (Christmann et al., 2023).

To address the problem of high adsorption of GarQ to biomass of the envelope of producer bacteria, we optimized medium composition by supplementation with CaCl_2_ and Tween 80. For nisin Z it was shown that addition of CaCl_2_ to the cultivation broth stimulates bacteriocin production in *L. lactis* (Matsusaki et al., 1996). The authors hypothesized that Ca^2+^ displaces cell adsorbed nisin. For *C. glutamicum*, it was shown that supplementation with CaCl_2_ results in increased production of secreted GFP (Teramoto et al., 2011; Freier et al., 2016) and Freier et al. (2016) hypothesized that CaCl_2_ might reduce electrostatic interaction of GFP and *C. glutamicum* cells by neutralizing the negative charges of the cell envelope. Further, supplementation with CaCl_2_ decreased nisin susceptibility and increased recombinant pediocin PA-1 production of *C. glutamicum* (Weixler et al., 2022; Christmann et al., 2023). In line with these findings, supplementation with CaCl_2_ led to a small decrease in adsorption of GarQ and resulted in a 2-fold increase in GarQ production by *C. glutamicum*.

Detergents are commonly used for solubilization and purification of amphiphilic membrane proteins (Linke, 2009; Duquesne and Sturgis, 2010) and the non-ionic detergent Tween 80 is described to reduce adsorption and increase production of bacteriocins (Garver and Muriana, 1994; Huot et al., 1996; Aymerich et al., 2000; Keren et al., 2004). We found that Tween 80 reduces adsorption of GarQ to *C. glutamicum* and, in combination, with CaCl_2_ increases titers of GarQ in supernatants of our *C. glutamicum* producer strains. Tween 80 also boosted GarQ activity and stabilized activity at neutral to alkaline pH in *in vitro* assays. An increase of bacteriocin activity in the presence of Tween 80 was also observed for lacticin RM (Keren et al., 2004). The stabilizing effect of Tween 80 on bacteriocin activity at neutral pH might be attributed to increased solubility of the peptide.

It is possible that CaCl_2_ and/or Tween 80 positively impact on expression of GarQ leading to increased levels of active GarQ in supernatants. However, our expression platform in *C. glutamicum* consists of an IPTG-inducible promoter (P_tac_) on a widely used plasmid (pXMJ19). Teramoto et al. have used a similar plasmid and the same IPTG-inducible promoter to show that CaCl_2_ at 2 g/L positively affects secretion of GFP (and amylase) by *C. glutamicum* but had no effect on *gfp* transcription or levels of cytosolic GFP (Teramoto et al., 2011). Thus, an effect of CaCl_2_ on *garQ* transcription is rather unlikely. Similar data on Tween 80 is, to our knowledge not available. To conclusively rule out effects of Tween 80 alone or in combination with CaCl_2_ on expression of GarQ, more comprehensive analyses by transcriptomics and proteomics are required.

Although *C. glutamicum* is considered to have low extracellular protease activity (Vertès, 2013; Freudl, 2018), several studies have shown that recombinant protein production by *C. glutamicum* can be improved by deleting proteases (Peng et al., 2019; Zhang et al., 2019; Liu et al., 2022). In a recent study, we investigated a possible contribution of extracellular protease activity to reduced GarQ levels during production with *C. glutamicum* (Desiderato et al., 2022). For these experiments, the *C. glutamicum* CR099 was grown over night in standard 2xTY medium. The conditions used for recombinant production GarQ production in the present study are, however, completely different, i.e., CGXII-U medium and acidic pH of the culture. A protease previously associated with Sec-dependent secretion stress in *C. glutamicum* is HtrA (Jurischka et al., 2020). HtrA proteases are widely distributed in pro- and eukaryotes and are quality control chaperones that play important roles during heat stress, pH stress and virulence (Hansen and Hilgenfeld, 2013; Backert et al., 2018). In *L. monocytogenes* and *Helicobacter pylori*, expression of HtrA is increased at low pH (Merrell et al., 2003; Stack et al., 2005). In line with these observations, we found increased levels of HtrA in *C. glutamicum* during cultivation under conditions of GarQ production in CGXII-U at acidic pH. More importantly, employing *htrA*-deficient *C. glutamicum* K9 for production instead of *C. glutamicum* CR099 resulted in increased GarQ titers (Figure 4). Similar observation were reported in *L. lactis* and *E. coli* showing that deletion of HtrA proteases improved recombinant protein production and stability (Baneyx and Georgiou, 1991; Meerman and Georgiou, 1994; Miyoshi et al., 2002; Cortes-Perez et al., 2006). It is quite possible that HtrA of *C. glutamicum* is involved in maintaining protein quality at low pH.

Another obstacle for production or storage described for pediocin PA-1 is the loss of activity by oxidation of a methionine residue (Johnsen et al., 2000). We previously observed that titers of active pediocin PA-1 are increased during recombinant production at low levels of dissolved oxygen in bioreactors or reduced aeration in non-baffled flasks (Goldbeck et al., 2021; Christmann et al., 2023). Similarly, cultivation of *C. glutamicum* K9/pXMJ19-SP*_*ywaD*_*-*garQ* in non-baffled flasks was beneficial for GarQ production. Of note, the natural producer *L. petauri* B1726 shows high GarQ production during cultivation at static conditions (Desiderato et al., 2022). Wildtype GarQ contains a methionine in position 5 of the active peptide. Loss in activity of GarQ incubated O/N with aeration (Supplementary Figure 5) and increased activity of the GarQ^M5F^ compared to GarQ (Figure 6A) support the notion that methionine oxidation may be involved in reduced GarQ in supernatants of *C. glutamicum* producers at high oxygen levels. However, loss of activity of GarQ^M5F^ after O/N incubation with aeration was even more pronounced than for wildtype GarQ (Figure 6B), and reduced activity at the end of fermentations in baffled flasks was also observed for GarQ^M5F^ (Figure 7B). This points toward other reasons for loss in activity or product under conditions with high aeration. In general, shaking of protein solutions generates air/liquid interfaces and hydrophobic interactions at these interfaces can lead to protein aggregation (Wang et al., 2010). It is possible that GarQ/GarQ^M5F^ are adsorbed to the surface of the glassware used for experiments by hydrophobic interactions. Also, vigorous shaking in baffled flasks may cause aggregation of the hydrophobic peptides GarQ and GarQ^M5F^. This would explain why activity remains on a high level in fermentation in non-baffled flasks. Similar observations were made for production of a secreted hydrophobic protein with *Pichia pastoris* (Woo et al., 2006). Here, aggregation of the protein was minimized by reducing stirrer speed and area of air/liquid interface.

Previously, we have shown successful production of other bacteriocins with *C. glutamicum* including pediocin PA-1, another bacteriocin that uses the PTS^Man^ as a receptor. We have also tried to the class I bacteriocin nisin. However, *C. glutamicum* is highly sensitive to nisin and a number of approaches to increase nisin resistance have failed (Weixler et al., 2021). Thus, we established a two-step process with production of the inactive precursor of nisin, which is then activated during downstream processing (Weixler et al., 2022). Further research is aimed at extending the range of bacteriocins (and other bioactive peptides) that can be produced wit *C. glutamicum*. However, the strategy for production has to be developed for each new product according to the level of resistance of *C. glutamicum*.

In summary, we show for the first time recombinant production of a bacteriocin by *C. glutamicum* using its native Sec translocon. By optimizing media and cultivation conditions and deletion of a quality control protease induced by pH stress, production and secretion of GarQ by *C. glutamicum* were improved to over 100 mg L^–1^. This is a 50-fold increase to GarQ levels obtained with a previously published first generation *C. glutamicum* producer strain employing the GarCD transporter. Although this is still 2-fold lower than GarQ levels obtained with the native producer *Lactococcus garvieae* production with *C. glutamicum* offers several advantages. On the one hand, natural producers are BSL2 pathogens while *C. glutamicum* is a biotechnological workhorse organism and generally recognized as safe. On the other hand, natural producers require rich, complex media to support growth whereas production processes with *C. glutamicum* on defined minimal media that allow downstream purification in pharmaceutical grade are well established. We also elucidate a number of factors that limit production of GarQ by *C. glutamicum*. This includes extracellular pH, HtrA protease activity, oxygenation, and, possibly, peptide aggregation. As these factors are not specific for GarQ but are rather related to general features of bacteriocins and/or associated with the production host, our findings may be of relevance for production of other bacteriocins using natural or recombinant producers.

## Data availability statement

The datasets presented in this study can be found in online repositories. The names of the repository/repositories and accession number(s) can be found below: ProteomeXchange Consortium–PXD046499.

## Author contributions

CD: Data curation, Investigation, Visualization, Writing—original draft, Writing—review and editing, Formal analysis. CM: Formal analysis, Investigation, Writing—review and editing, Methodology. AS: Formal analysis, Investigation, Writing—review and editing. BG: Formal analysis, Investigation, Writing—review and editing. KH: Formal analysis, Investigation, Writing—review and editing. BS: Formal analysis, Investigation, Writing—review and editing. VS: Formal analysis, Investigation, Methodology, Writing—review and editing. AR: Formal analysis, Investigation, Methodology, Writing—review and editing. MO: Formal analysis, Methodology, Writing—review and editing, Conceptualization, Funding acquisition, Project administration, Resources, Supervision. BE: Conceptualization, Formal analysis, Funding acquisition, Methodology, Project administration, Resources, Supervision, Writing—review and editing. CR: Conceptualization, Funding acquisition, Methodology, Project administration, Resources, Supervision, Writing—review and editing, Data curation, Investigation, Visualization, Writing—original draft.
