# Supplementary material for: Optimized recombinant production of the bacteriocin garvicin Q by Corynebacterium glutamicum
Source: Front Microbiol. 2024 Jan 8;14:1254882. doi: 10.3389/fmicb.2023.1254882 (PMC10800739; doi:10.3389/fmicb.2023.1254882)
Supplement: Supplementary file 1 [file Data_Sheet_1.PDF]

Supplementary Material

Optimized recombinant production of the bacteriocin garvicin Q by *Corynebacterium glutamicum*

Christian K. Desiderato[1], Carolin Müller[2,3], Alexander Schretzmeier[1], Katharina M. Hasenauer[1], Bruno Gnannt[1], Bastian Süpple[1], Alexander Reiter[2,3], Valentin Steier[2,3], Marco Oldiges[2,3], Bernhard J. Eikmanns[1], Christian U. Riedel[1,*]

*** Correspondence:** christian.riedel@uni-ulm.de

1 Supplementary Figures and Tables

1.1 Supplementary Figures

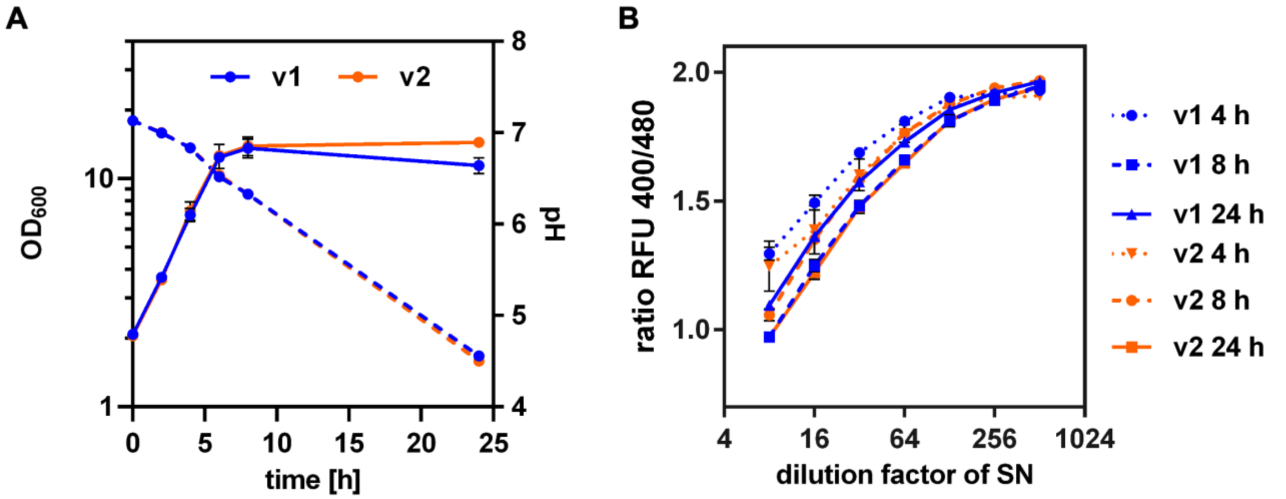

Figure S1: Comparison of *C. glutamicum* CR099/pXMJ19-SP*ywaD*-*garQ*-v1 and *C. glutamicum* CR099/pXMJ19-SP*ywaD*-*garQ*-v2 differing in the presence (v1) or absence (v2) of an additional two amino acids at the N-terminus of GarQ following cleavage by the Sec signal peptidase. (A) Growth (OD_{600}) and (B) antimicrobial activity in culture supernatants of the two strains grown in CGXII-U at the indicated time points during cultivation. Antimicrobial activity of recombinant GarQ was determined by pHluorin2 assay using *L. lactis* IL1403/pNZ-pHin2^{Lm} as indicator strain. Values are ratios of fluorescence intensity of the biosensor (emission at 520 nm) after excitation at 400 and 480 nm (ratio RFU 400/480). All values are mean ± standard deviation of three independent experiments, i.e. supernatants of independent cultivations per strain.

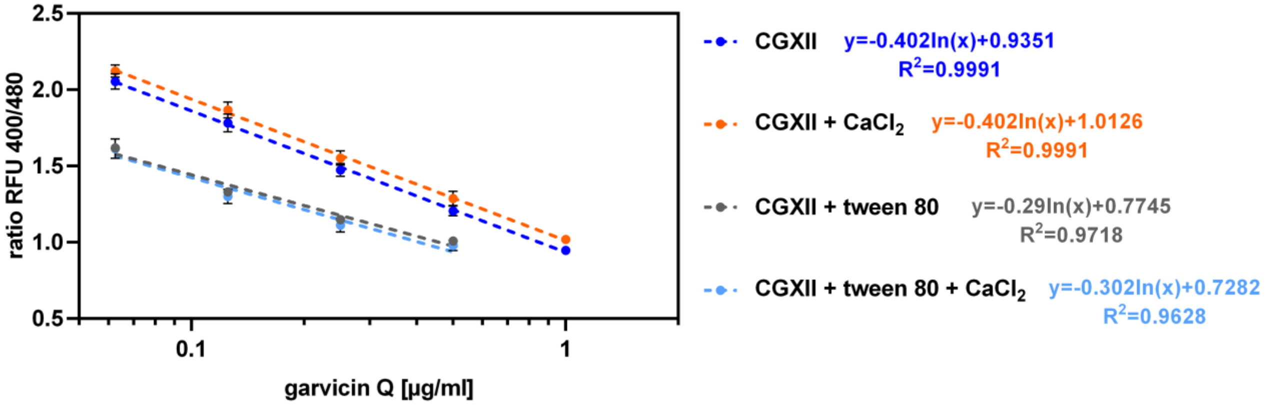

Figure S2: Calibration for quantification of GarQ in different media using results of pHluorin2 assays. The sensor strain *L. lactis* IL1403/pNZ-*pHin2*^{Lm} was incubated in standards of chemically synthesized GarQ in CGXII-U medium with the indicated supplements (0.5 g l^{-1} $CaCl_2$ and/or 0.5% (w/v) Tween 80) for 30 min and ratios of fluorescence intensities of the biosensor (emission at 520 nm) were determined with excitation at 400 and 480 nm (ratio RFU 400/480). GarQ standard was prepared and measured in triplicates for all conditions. Calibration curves for GarQ concentration as a function of the mean of ratios RFU 400/480 were established for each condition by calculating a logarithmic fit. The equation of the calibration curves can be used to calculate mass concentrations of active GarQ in these media.

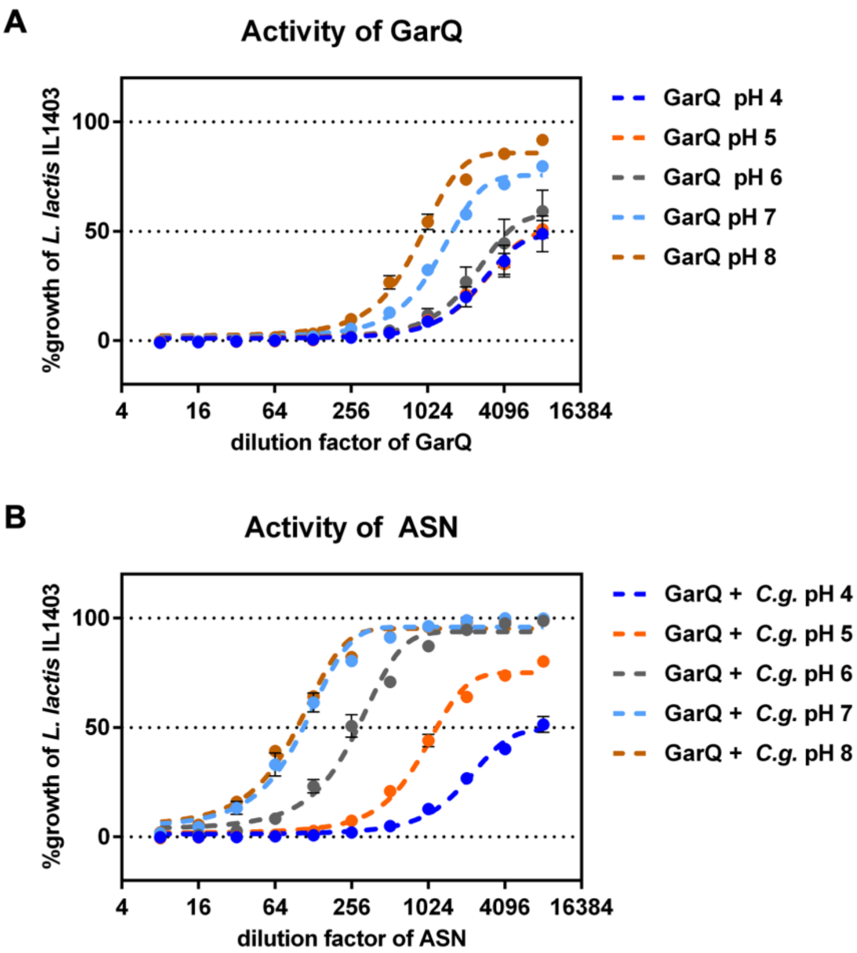

Figure S3: Extracellular pH affects adsorption of GarQ. HIC-purified GarQ was incubated alone or with *C. glutamicum* CR099 cells for 1 h at 30 °C in UB3 buffer at the indicated pH. After incubation, bacteria were pelleted by centrifugation and the adsorption supernatant (ASN) was collected. Activity of serial two-fold dilutions of GarQ (A) or ASN (B) was determined by growth inhibition assays using *L. lactis* IL1403/pNZ-pHin2^{Lm} as sensor. Values are mean ± standard deviation of n = 3 independent experiments. For each curve, data points were fitted by a nonlinear fit based on the Gompertz function using GraphPad Prism 6 software. The coefficient of determination (R^2) of the Gompertz function was above 0.97 in all cases, indicating that the function is suitable. Arbitrary units (AU; in Table 2) of bacteriocin activity of samples are defined as the X-value of the inflection point (1/K) of the Gompertz function.

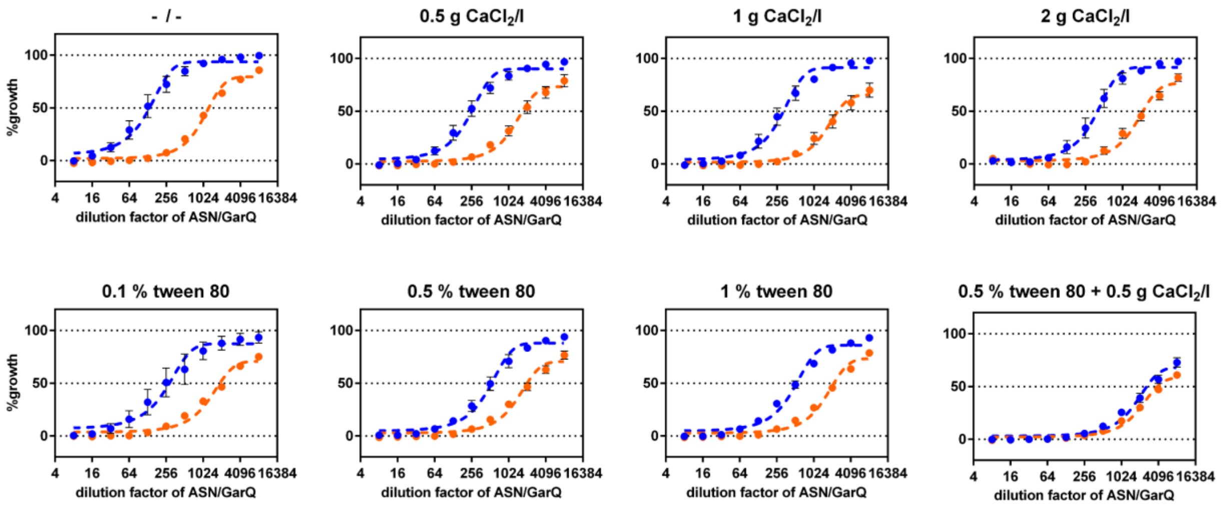

Figure S4: $CaCl_2$ and Tween 80 affect adsorption of GarQ to *C. glutamicum* biomass. HIC-purified GarQ was incubated alone or with *C. glutamicum* CR099 cells for 1 h at 30 °C in UB3 buffer with the indicated concentrations of $CaCl_2$ and/or Tween 80. After incubation, bacteria were pelleted by centrifugation and the adsorption supernatant (ASN) was collected. Activity of serial two-fold dilutions of GarQ incubated without bacteria (orange) or ASN (blue) was determined by growth inhibition assays using of *L. lactis* IL1403/pNZ-pHin2^{Lm} as sensor. Values are mean ± standard deviation of n = 3 independent experiments. For each curve, data points were fitted by a nonlinear fit based on the Gompertz function using GraphPad Prism 6 software. The coefficient of determination (R^2) of the Gompertz function was above 0.97 in all cases, indicating that the function is suitable. Arbitrary units (AU; in Table 4) of bacteriocin activity of samples are defined as the X-value of the inflection point (1/K) of the Gompertz function.

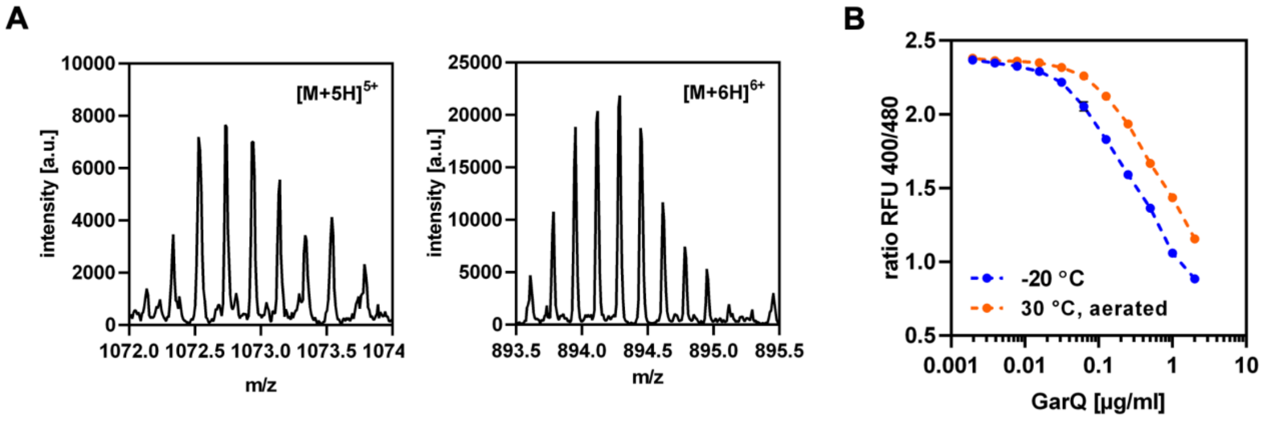

Figure S5: Oxidized GarQ is present in supernatants of *C. glutamicum* CR099/pXMJ19-SP$_{ywaD}$-*garQ*-v2 grown in CGXII-U and incubation of GarQ in aerated conditions reduces activity. (A) LC–MS spectra of combined RPC elution fraction harboring antimicrobial activity zoomed on the peaks of oxidized garvicin Q (5356.63 Da) carrying five (5: $[M+5H]^{5+}$, m/z = 1072.326, m/z spacing: 0.200) and six (6: $[M+6H]^{6+}$, m/z = 893.772, m/z spacing: 0.167) positive charges. (B) Activity of serial two-fold dilutions of GarQ incubated o/N at 30° with aeration on a rotary shaker (orange) or stored at -20 °C (blue) was determined by pHluorin2 assays using *L. lactis* IL1403/pNZ-pHin2^{Lm} as sensor. Values are ratios of fluorescence intensity (emission at 520 nm) of the biosensor after excitation at 400 and 480 nm (ratio RFU 400/480) and are mean ± standard deviation (SD) of three independent experiments.

1.2 Supplementary Tables

Table S1: Primer sequences used in present study.

Primer	Sequence (5´->3´)	Purpose
garQ-GGA-fwd	AGATAGGTCTCGAATTCGAATACCACCTGATGAATGGTG	Amplification of *garQ* for Golden Gate Assembly
garQ-GGA-rev	AGATAGGTCTCGATAAGTTAATGTTGAGGGCCAAAC	
ywaD-PstI-fwd	AGACTGCAGAGGAGAAAAACATATGAAGA	Construction of pXMJ19-*ywaDSPgarQ* v1
garQSP-BamHI-rev	AGAGGATCCTTAATGTTGAGGGCCA	
ywaDSP_fwd	ATTAAGCTTGCATGCCTGCA GAGGAGAAAAACATATGAAGAAGCTGCTGACC	Construction of pXMJ19-*ywaDSPgarQ* v2
ywaDSP_rev	GGTGGTATTCTGCGTGTGCTGCTGGGGT	
garQ_fwd	AGCACACGCAGAATACCACCTGATGAATG	
garQ_rev	ATTCGAGCTCGGTACCCGGGTTAATGTTGAGGGCCAAAC	
